# Identification of 20-Hydroxyecdysone Late-Response Genes in the Chitin Biosynthesis Pathway

**DOI:** 10.1371/journal.pone.0014058

**Published:** 2010-11-18

**Authors:** Qiong Yao, Daowei Zhang, Bin Tang, Jie Chen, Jing Chen, Liang Lu, Wenqing Zhang

**Affiliations:** 1 State Key Laboratory of Biocontrol, School of Life Sciences, Sun Yat-sen University, Guangzhou, China; 2 Hangzhou Key Laboratory of Animal Adaptation and Evolution, Hangzhou Normal University, Hangzhou, China; University of Kent, United Kingdom

## Abstract

**Background:**

20-hydroxyecdysone (20E) and its receptor complex ecdysone receptor (EcR) and ultraspiracle (USP) play a crucial role in controlling development, metamorphosis, reproduction and diapause. The ligand-receptor complex 20E-EcR/USP directly activates a small set of early-response genes and a much larger set of late-response genes. However, ecdysone-responsive genes have not been previously characterized in the context of insect chitin biosynthesis.

**Principal Findings:**

Here, we show that injection-based RNA interference (RNAi) directed towards a common region of the two isoforms of *SeEcR* in a lepidopteron insect *Spodoptera exigua* was effective, with phenotypes including a high mortality prior to pupation and developmental defects. After gene specific RNAi, chitin contents in the cuticle of an abnormal larva significantly decreased. The expression levels of five genes in the chitin biosynthesis pathway, *SeTre-1*, *SeG6PI*, *SeUAP*, *SeCHSA* and *SeCHSB*, were significantly reduced, while there was no difference in the expression of *SeTre-2* prior to 72 hr after injection of *EcR* dsRNA. Meanwhile, injection of 20E *in vivo* induced the expression of the five genes mentioned above. Moreover, the *SeTre-1*, *SeG6PI*, *SeUAP* and *SeCHSB* genes showed late responses to the hormone and the induction of *SeTre-1*, *SeG6PI*, *SeUAP* and *SeCHSB* genes by 20E were able to be inhibited by the protein synthesis inhibitor cycloheximide *in vitro* indicating these genes are 20E late-response genes.

**Conclusions:**

We conclude that *SeTre-1*, *SeG6PI*, *SeUAP* and *SeCHSB* in the chitin biosynthesis pathway are 20E late-response genes and 20E and its specific receptors plays a key role in the regulation of chitin biosynthesis via inducing their expression.

## Introduction

Throughout the insect life cycle, the steroid hormone 20-hydroxyecdysone (20E) coordinates multiple developmental events by eliciting a complex genetic program via a heterodimeric nuclear receptor composed of the ecdysone receptor (EcR) and ultraspiracle proteins (USP). Evidenced in a study of fruit fly *Drosophila melanogaster* by Ashburner *et al.* first revealed that a part of this developmental program consists of a genetic cascade in which the ligand-receptor complex 20E-EcR/USP directly activates the expression of a very small number of early-response genes. The products of the early-response genes in turn trigger the expression of a much larger set of late-response genes, so called secondary-response genes [1], [2], [3]. During the last decade, the *EcR*, *USP* and a large number of ecdysone-responsive genes from the two gene categories proposed in the Ashburner model have been characterized in *D. melanogaster* and several other insect species [4], [5], [6]. All of those studies published over the past decade have provided powerful evidence in support of the Ashburner model, meantime taken beyond that model which presented us with diverting new directions for our understanding of ecdysone signaling [7], [8], [9], [10].

Most of the previous studies are extensively emphasize DNA puffs induced by ecdysone, whereas a few specific DNA puff genes are studied for a better understanding of the mechanisms underlying the ecdysone response on molecular level. The roles and functions of many early-response genes (eg. *E74*, *E75* and *broad-complex*) have been well studied [11], [12], while much less is known about the late-response genes (*L63*, *L71* and *L82*) in insects. L63 has homology to the cyclin dependent kinase protein family and is required for *Drosophila* development [13]; *L71* genes encode a set of polypeptides and provide an antimicrobial defense during metamorphosis [14]. *L82* mutations displayed developmental delay and eclosion lethal phenotypes in *Drosophila* [15]. The late genes play direct or indirect roles and also a distinct role in controlling the appropriate biological response to hormone, including development, metamorphosis, reproduction and diapause. As outlined above, most of the studies regarding the ecdysone-responsive genes in insects have focused on the regulatory genes at the top of the ecdysone-elicit genetic hierarchy.

**Figure 1:**
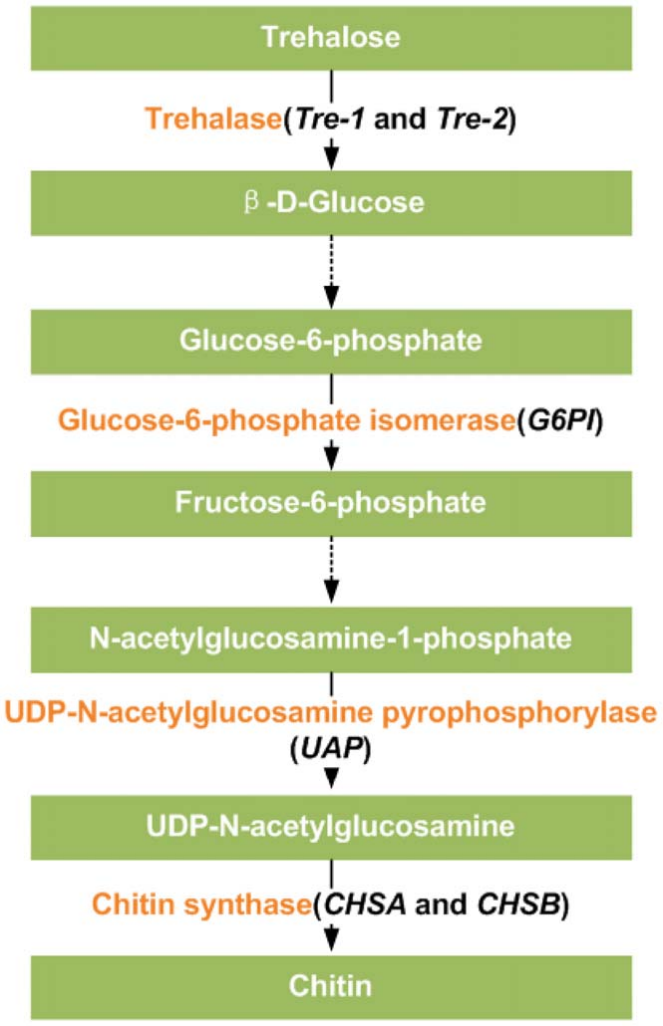
A brief diagram of insect chitin biosynthesis pathway. The black italics in parenthesis indicate six genes encoding the enzymes.

Chitin is the major polysaccharide layed in the cuticle, peritrophic matrix, tracheae and muscle attachment points as a characteristic constituent of insects and other arthropods. The chitin biosynthesis pathway begins with glycogen and trehalose, and consists of a patchwork of at least eight key enzymes (Fig. 1) [16]. Importantly, the process of chitin biosynthesis and degradation is strictly coordinated within the cycle of molts and behaves as an ecdysone-induced response [17], [18], [19]. Many studies have demonstrated the participation of 20E-EcR/USP complex in regulation of gene expression; however, the ecdysone-responsive genes involved in the process of chitin biosynthesis are still largely unclear.

Here we cloned two *EcR* isoforms, *SeEcR-A* and *SeEcR-B1*, from the beet armyworm, *Spodoptera exigua*, a wide-spread, destructive and polyphagous noctuid lepidopteron pest. Consequently, we confirmed that the injection-based RNAi of *SeEcR* leads to a delay in developmental duration, reduced food intake, kinds of defect phenotypes in pupae formation and adults eclosion, and also chitin content reduction in the cuticle of abnormal larvae. The effects of RNAi on the target gene were proved to be gene-specific and effective, with the efficiency lasting for 108 hr. The results after the injection of dsRNA for *EcR* or 20E *in vivo* and the midgut culture experiments *in vitro* revealed that the regulation of *EcR* remarkably affects the expression of five genes (*SeTre-1*, *SeG6PI*, *SeUAP*, *SeCHSA* and *SeCHSB*) in the chitin biosynthesis pathway, and genes encoding SeTre-1, SeG6PI ,SeUAP and SeCHSB were identified as 20E late-response genes.

## Results

### Isolation and sequence analysis of EcR-A and EcR-B1 cDNAs

**Figure 2:**
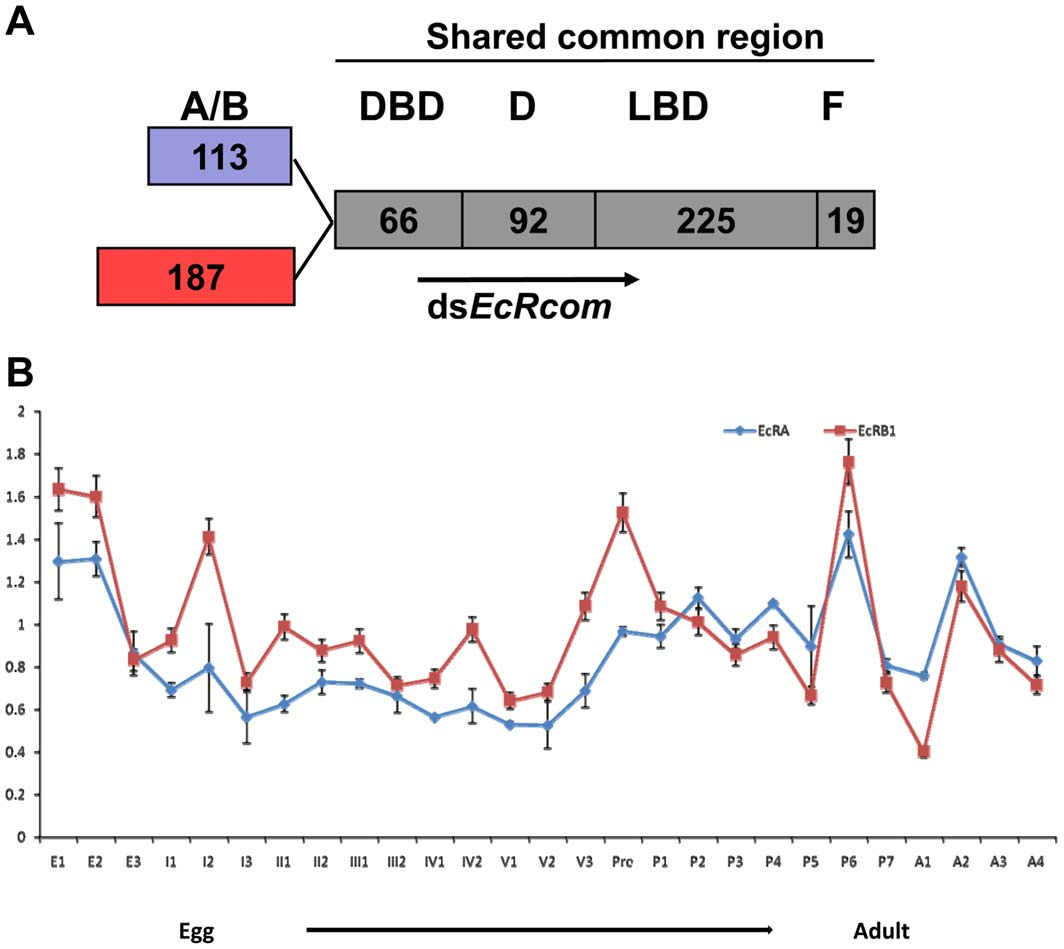
The domain structures and the developmental expression pattern of the two EcR isoforms in *S. exigua*. (A) The domain structures of SeEcR-A and SeEcR-B1 and the common fragment used to generate the dsRNAs. The number of amino acids of each domain is indicated. (B) The developmental expression profiles of *SeEcR-A* and *SeEcR-B1* mRNA. Total RNAs were prepared from individuals of *S. exigua* collected at all developmental stages from the first day of eggs to the last day of adults. The levels of the mRNA were detected using qRT-PCR. The mRNA level of β-Acitn was used as a control to normalize for loading. The data represent the mean values ± SE of three replicates. The age of the insects is indicated in: E1, the first day of eggs; I1, the first day of the first instar larvae; pre, prepupal period; P1, the first day of pupae; A1, the first day of adults.

Cloning of *EcR* cDNAs from *S. exigua* was accomplished by RT-PCR using degenerate primers designed on the basis of conserved motifs in the DNA binding domain (DBD) and ligand binding domain (LBD) of insect *EcR* sequences. To subclone, cDNA from the first instar larvae was used as a template to obtain a 527-bp PCR fragment of the shared common domain, and two full-length cDNAs were obtained with a combination of 3′-RACE and 5′-RACE methodologies. Database BLAST search revealed that these cDNAs encoded *S. exigua* homologs of the nuclear receptor EcR, which contain a common carboxy-terminal region with a DNA-binding and ligand-binding domains but unique amino-termini of 113 and 187 amino acids (Fig. 2A). The two cDNAs were sequenced with an open reading frame of 1546 and 1767 nucleotides, encoding a protein of 514 and 588 amino acids with a calculated molecular weight of 57.4 and 66.0 kDa, respectively.

Hence, on the basis of the similarity in their A/B domains when aligned with other insect EcR members, one cloned cDNA encodes an EcR-A isoform with a conserved 20 amino acid sequence (specific A-box) at the C-terminal of the A/B domain of all isoform-A receptors, and the other one is a SeEcR-B1 isoform. The deduced amino acid sequences of the two isoforms showed high similarity to the reported insect *EcR* sequences. *SeEcR-A* (GenBank accession number: GU296540) is 90% identical to *SeEcR-B1* (GenBank accession number: EU426551).

By quantitative real-time PCR, the developmental expression patterns of *SeEcR-A* and *SeEcR-B1* were investigated in all developmental stages including the first day of egg to the last day of adult. The results showed that both *SeEcR-A* and *SeEcR-B1* were continuously expressed through the whole life cycle of *S.exigua* in correlation with the ecdysteroid pulse. (Fig. 2B).

### The efficiency of RNAi towards the two EcR isoforms

**Figure 3:**
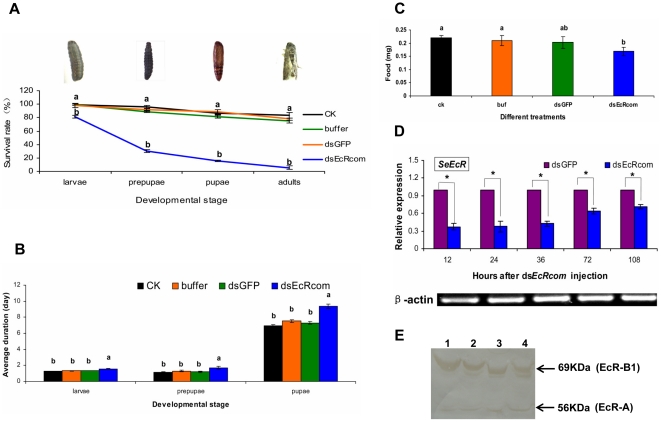
The survival rates, developmental duration, food-intake and the changes of SeEcR mRNA and protein in *S. exigua* after *SeEcR* RNAi. (A) The accumulated percentage of survival rates, (B) Developmental duration, (C) Food intake, (D) The relative expression level of *SeEcR* and (E) The SeEcR protein level of *S.exigua* larvae in different treatments. For (A, B, C), the following three controls were included: an equivalent amount of *GFP* dsRNA, the same volume of buffer group (DEPC water) and the CK control group (no treatment). Data are presented as mean ± SE from three independent experiments with 30 fifth-instar larvae in each group. The lowercase letter “a” at each developmental stage in Fig. 3A represents no significant difference among ds*GFP*, buffer and CK groups but the survival rates in all three control groups are significantly different from that in the ds*EcRcom*-injection group (labeled with a lowercase “b” at the same developmental stage). Different letters at the same developmental stage in Fig. 3B and Fig. 3C among different treatments indicate significant differences of either developmental duration or food-intake (p<0.05, Duncan multiple comparison test, SPSS). For (D), the expression level of *SeEcR* and *Seβ-actin* transcripts were detected using qRT-PCR. By definition, the mRNA level of the target gene was normalized to *Seβ-actin* in each group; and the relative expression level of the target gene was measured as the level in the ds*EcRcom*-injection group divided by the level in the ds*GFP*-injection group. Data are shown as mean ± SE from three independent experiments. An asterisk indicates significant differences in the expression level between the treated and control groups measured at the same time (p<0.05, T test). For (E), the protein levels of target genes were detected using Western blot. A total of 80 ug of proteins from each individual treated with ds*GFP* or ds*EcRcom* was applied to each lane and separated by 10% SDS-polyacrylamide gel electrophoresis. Lanes 1 and 3: 36 and 12 hours post-ds*GFP* injection; Lanes 2 and 4: 36 and 12 hours post- ds*EcRcom* injection, respectively. The results are representative of four replicates.

To analyze whether it was possible to reduce *SeEcR* levels by RNAi *in vivo*, a 546 bp fragment containing the coding region shared by the two *EcR* isoforms, named as ds*EcRcom* was used for dsRNA synthesis (Fig. 2A). qRT-PCR results showed that *SeEcR* mRNA levels clearly decreased in the ds*EcRcom*-injected insects as compared with those in the ds*GFP*-injected control (Fig. 3D). At 12 hours post-treatment, ds*EcRcom* injection resulted in an approximately 3-fold knock-down of the *SeEcR* transcript level. Moreover, in western blot analysis result, the protein level changes of the target genes showed that both SeEcR-A and SeEcR-B1 were not influenced at 12 hours after dsRNA injection, but decreased immune signals are detected in response to the ds*EcRcom* injection at 36 hours post-injection (Fig. 3E). Additionally, the silencing effect on the target gene lasted for as long as 108 hr, suggesting that the microinjection of dsRNA was effective at silencing the *EcR* genes in *S. exigua*.

**Figure 4:**
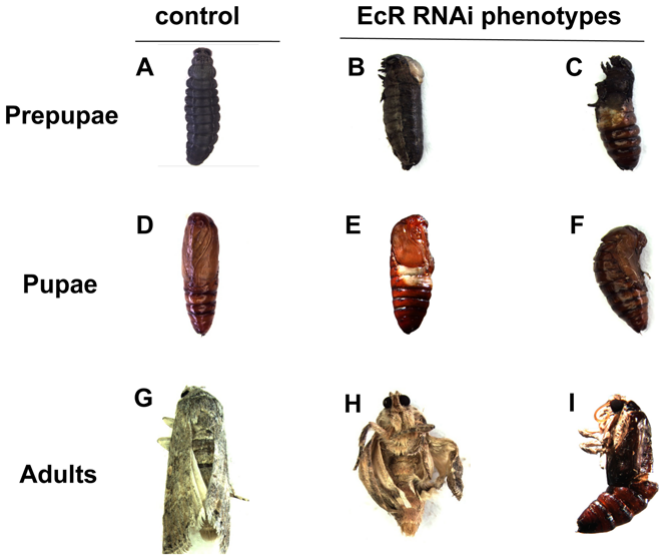
Lethal phenotypes caused by ds*EcRcom* injection into fifth-instar larvae. (A, D, G) Normal prepupa, pupa and adult (B, C) Abnormal prepupae (E, F) Severe misshaped pupae, including typical “half pupation” pupa (E) and “Abnormal eclosion” adults (H, I).

We observed severe developmental abnormity and phenotypic defects caused by the dramatic changes in the mRNA level of *SeEcR* by *SeEcR*-specific RNAi in *S. exigua*. Quite remarkably, following ds*EcRcom* injection, the average survival rates were 81.11%, 30.00%, 15.56%, and 5.56% in the fifth-instar larvae, prepupae, pupae and adults, respectively, and were significantly lower than those in the three controls (Fig. 3A). A sharp decline in survival rate was observed between larva-prepupa metamorphosis after RNAi. In the ds*EcRcom*-injected insect, pupation was abnormal and the pupa–adult metamorphasis could not complete, with the pupal exocuticle partially shelled, the adult in pupal cuticle arrested entrapped or the wing brokenly extended. Although the severity of the phenotype defects varied somewhat, two typical severe defect phenotypes, “half pupation” larvae and “abnormal eclosion” adults, were observed (Fig. 4B, C, H, I). These “half pupation” larvae, which were not observed in the controls, always failed to complete pupation and died within one or two days. Moreover, the ds*EcRcom*-injected group also had evidently longer developmental durations in the larva, prepupa and pupa phase (Fig. 3B). In contrast, no significant lethality differences were found among the three controls. In addition, many individuals injected with ds*EcRcom* shrank in body size and had reduced food-intake (Fig. 3C).

### Chitin levels in the epidermis and midgut/peritrophic membrane after RNAi

To address the impact of lower *SeEcR* transcript levels on the chitin metabolism, the chitin contents in the epidermis and the midgut/peritrophic membrane (PM) were determined. From 12 hr to 36 hr post injection of dsRNA, the chitin contents in both the midgut/PM and the epidermis decreased in both treatments of *dsGFP* and *dsEcRcom* (Table 1). At 12 hr post injection, no significant difference on chitin levels was found between treatments of *dsGFP* and *dsEcRcom*. At 36 hr post injection, however, the chitin content of the midgut/PM in the ds*GFP* treatment group was relatively lower than that in the ds*EcRcom* treatment group. Since chitin degradation starts in the midgut/PM in this stage [18], [20], the reduction in *SeEcR* levels may result in slow chitin degradation carried out in the midgut/PM. While in the epidermis at 36 hr post injection, both degradation of larvae epidermis chitin and generation of pupae cuticle chitin happened in prepupal stage. Lower levels of *SeECR* may have different effects on the degradation and the generation of the chitin, which may result in relatively higher chitin content in the ds*GFP* treatment. These results were also in correlation with the longer developmental duration observed in this study.

**Table 1:** The effect of injecting *EcR* dsRNAs on chitin content of different tissues in *S. exigua* larvae.

Tissues	Group	Mean (µg chitin/insect) ± SE	
		12 hr	36 hr
Midgut/peritrophic membrane	ds*GFP*	5.7219±0.7580 a	0.7094±0.0665 b
	ds*EcRcom*	5.6726±0.4949 a	1.9533±0.3530 a
Epidermis	ds*GFP*	39.7933±2.1478 a	33.1551±1.8894 a
	ds*EcRcom*	39.6455±2.9804 a	29.1587±1.5632 b

Different letters between treatments of ds*GFP* and ds*EcRcom* in the same column indicate significant difference in the chitin contents in either the midgut or the epidermis (p<0.05, T test, n = 8).

### Effects of *SeEcR* RNAi on the expression of key genes in the chitin biosynthesis pathway

The chitin analysis results (Table 1) suggested that *SeEcR* expression affected the chitin contents in the epidermis and the peritrophic membrane. Thus, six genes involved in the chitin biosynthesis pathway were examined to ascertain their responsiveness to ecdysone receptor expression using qRT-PCR.

**Figure 5:**
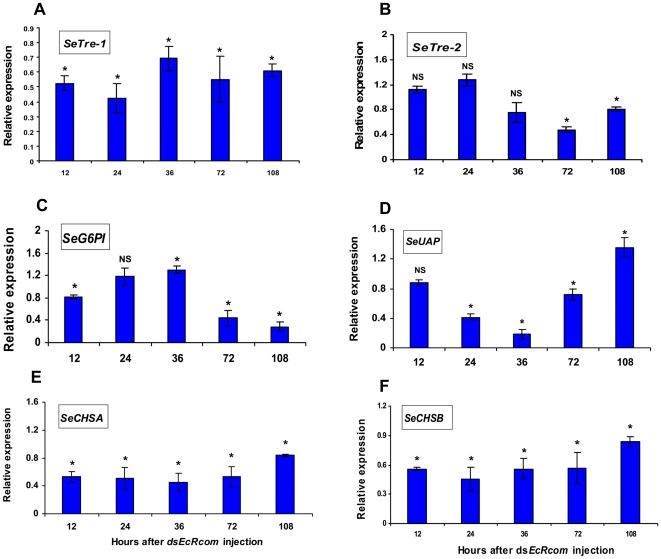
Changes in mRNA expression levels for key genes in the pathway following *SeEcR* RNAi. The expression levels of six transcripts (*SeTre-1, SeTre-2, SeG6PI, SeUAP, SeCHSA* and *SeCHSB*) were detected by qRT-PCR at five detection points after ds*EcRcom* injection. Data are represented as mean ± SE from three independent experiments, which were analyzed in the same way as in Fig. 3. *: a significant difference between the ds*EcRcom* injection group and the ds*GFP* injection group (p<0.05, T test); NS: no significant difference between the two groups.

Concurrently, at 12 hr after ds*EcRcom* injection, the expressions of *SeTre-1* (EU427311), *SeCHSA* (DQ062153) and *SeCHSB* (EU622827) were dramatically down-regulated in the ds*EcRcom*-injected larvae and reduced to approximately half of the mRNA levels in the control insects. Coinciding with the low expression level of *SeEcR*, the significantly suppressed effect on these three genes lasted for 108 hr (Fig. 5A, E and F). The *SeCHSA* and *SeCHSB* genes seem to share a similar down-regulation pattern. Meanwhile, *SeTre-2*, *SeG6PI* and *SeUAP* had extraordinary responses to *SeEcR* RNAi. The transcriptional abundance of *SeG6PI* (unpublished) was repressed at 12 hr after ds*EcRcom* injection, with a small rise at 36 hr, and then remained low until 108 hr post-injection of ds*EcRcom* (Fig. 5C). Quite differently, *SeUAP* (FJ380203) expression was slightly affected at the first testing point, and the repressed transcription level of *SeUAP* was only observed at 24 hr and 72 hr post-injection with a detectable but subtle increase in expression at 108 hr after ds*EcRcom* injection (Fig. 5D). Conversely, hardly any expressional variation of *SeTre-2* (EU106080) was observed before 72hr post-injection of ds*EcRcom* (Fig. 5B). The qRT-PCR results revealed different transcript level changes in the six genes involved in chitin biosynthesis, which suggests that the effects of *EcR* on the six genes varied somewhat.

### Influence of 20E on expression of SeEcR and key genes in the pathway

**Figure 6:**
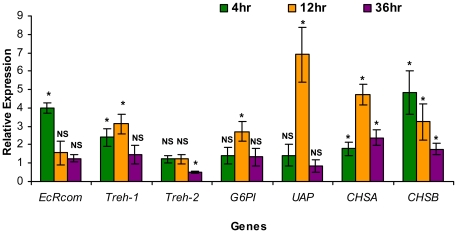
The influence of 20E on expression of *SeEcR* and key genes in the pathway. The mRNA expression levels of *SeEcR* and the six chitin biosynthesis pathway genes were detected using qRT-PCR at 4 hr, 12 hr and 36 hr after injection of 20E. Data are represented as mean ± SE from Six independent experiments which were analyzed in the same way as Fig. 3. *: significant difference between the ds*EcRcom* injection group and the ds*GFP* injection group (p<0.05, T test); NS: no significant difference between the two groups.

After 20E injection, the relative mRNA expressions of *SeEcR* and the six genes in the various groups of *S. exigua* larvae were assayed using qRT-PCR. The results showed that there was a burst in *SeEcR* transcription that increased nearly 4-fold when compared to the mRNA level in control insects at 4 hr after 20E injection, while there was no difference in the relative abundance at 12 hr and 36 hr after 20E injection as compared with the control (Fig. 6). At 12 hr post-injection of 20E, the mRNA expressions of *SeTre-1*, *SeG6PI* and *SeCHSB* showed about 3-fold up-regulation, *SeCHSA* transcript was up-regulated over 4-fold and *SeUAP* mRNA level was increased nearly 7-fold. Thereafter, a significantly stimulated up-regulation of the *SeCHSA* and *SeCHSB* genes were observed at 36 hr after 20E injection, synchronously coinciding with distinct expression of the *SeTre-1*, *SeG6PI* and *SeUAP* genes whose variation of transcript abundance were barely detectable at this detection point. On the contrary, treatment with 20E resulted in a delay in changes of the *SeTre-2* expression before 36 hr post-injection of 20E. Hence, the expression of the five genes (excluding *SeTre-2*) continued to increase in the 20E injection group, suggesting that these five genes are probably among the ecdysone-responsive genes.

### Response of genes in the pathway to ecdysone or cycloheximide *in vitro*

**Figure 7:**
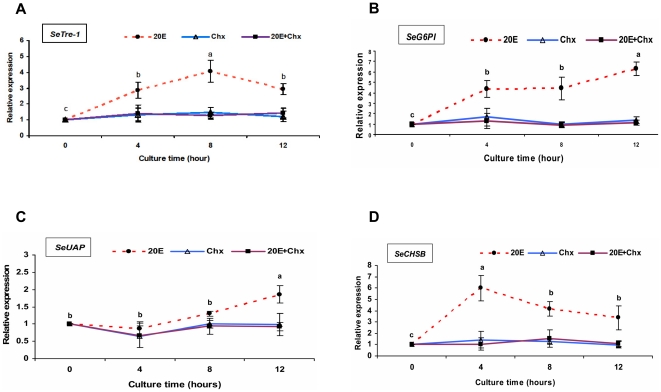
The effect of 20E and cycloheximide on the mRNA expression level of the key genes in midgut *in vitro* culture. The midguts were cultured with10^−6^ M 20E (20E), 10^−3^ M cycloheximide in the hormone-free medium (Chx), in the continuous presence of both 10^−6^ M 20E and10^−3^ M Chx (20E+Chx), or with no addition in the medium (control). The mRNA expression levels of four genes, including *SeTre-1* (A), *SeG6PI* (B), *SeUAP* (C) and *SeCHSB* (D), were determined using qRT- PCR after incubating for 0 hr, 4 hr, 8 hr and 12 hr. The expression level of each target gene was normalized to the *Seβ-actin* at each time point, then the mRNA levels in the three treatment groups were normalized to the control group at each time point. The relative expression level of each target gene (y-axis) was calculated as the mRNA level at each time divided by the level at 0 hr in each group. Data are represented as mean ± SE from three independent experiments. Different letters on the broken line (20E incubation group) indicate significant differences among different culture time points in this group. (p<0.05, Duncan multiple comparison test, SPSS).

To further determine the hierarchy position in the EcR/USP-20E-mediated genetic regulatory network, the transcript accumulation patterns of all genes (except *SeCHSA*, a non-midgut gene) in the pathway were investigated in cultured midguts *in vitro*. Incubation of the midguts in the presence of both 10^−6^ M 20E and 10^−3^ M cycloheximide (Chx,the protein synthesis inhibitor) in the medium slightly or hardly induced the following four genes: *SeTre-1*, *SeG6PI, SeUAP* and *SeCHSB*, a pattern quite similar to that in incubation with the same dose of Chx in the hormone-free medium as determined at p<0.05 using T test (Data not shown). As opposed to two treatments mentioned above, the four mRNA induction profiles in the cultured tissues incubated with 10^−6^ M 20E were siginificantly different at various times points (Fig. 7). When analyzing larval midguts cultured in the presence of 20E, the induced expression peaks of *SeTre-1* and *SeCHSB* were detected 4 hr later, and those of *SeG6PI* and *SeUAP* could be sensed 8 hr later (Fig. 7A, B, C, D). In this *in vivo* midgut culture experiment, the data demonstrated that the expression of these four genes could be induced by 20E but inhibited by Chx.

## Discussion

### Characteristics of *S. exigua* EcR isoforms and their role in developmental processes

**Figure 8:**
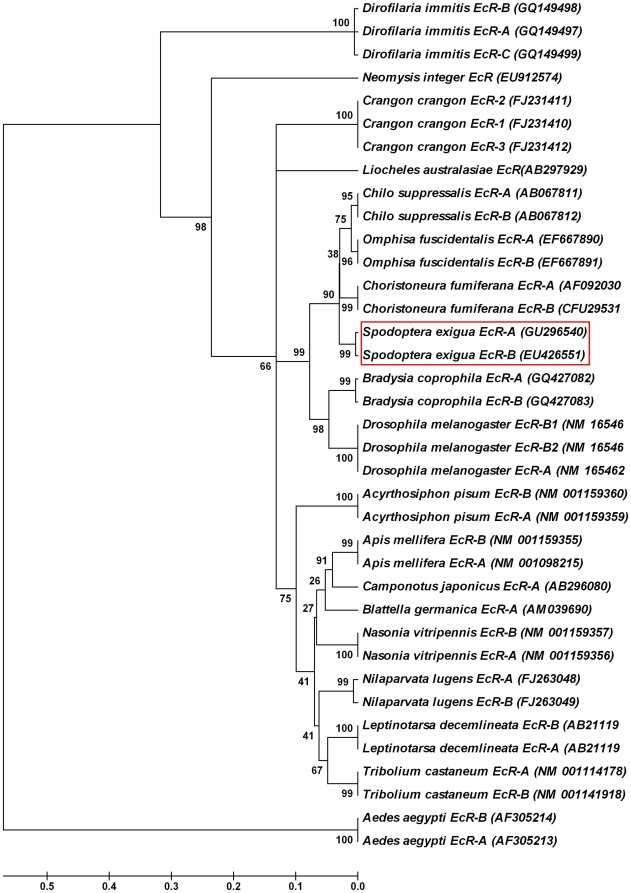
A phylogenic tree of *EcR-A and EcR-B* genes in insects and other organisms. A phylogenic tree was constructed using the Molecular Evolutionary Genetics Analysis (MEGA) software version 4.1 with the sequences obtained from GenBank. The robustness of each cluster was verified in 50,000 replicates. The scale on the x-axis represents the estimated branch lengths and numbers indicate the bootstrap values.

We cloned and characterized two *EcR* isoforms (Fig. 2). Analysis of the deduced amino acid sequences indicated that they were two isoforms of EcR, a member of the steroid hormone receptor superfamily, homologous to the *Drosophila* EcR (Fig. 8). The fact that the two isoforms have distinctive N-terminal A/B domain and share a highly conserved amino acid sequence in all other domains at the C-termini suggests they are splice variants generated from the same *EcR* gene transcript. Ever since the *Drosophila EcR* gene was cloned and the three EcR isoforms (*EcR-A*, *EcR-B1* and *EcR-B2*) were characterized [21], two isoforms of *EcR* (*EcR-A* and *EcR-B1*) were only reported in a few lepidopteron species, including *Bombyx mori*, *Choristoneura fumiferana* and *Chilo suppressalis* [22], [23], [24]. More evidence is needed to confirm whether three isoforms actually exist in lepidopteron insects.

The regulation of EcR on insect development has been well demonstrated by mutational analysis and by using inducible expression of double stranded RNA (dsRNA) of *EcR* isoforms in *D. melanogaster*, which resulted in a block in neuronal remodeling and ecdysone responses during larval molting and metamorphosis [25], [26], [27], developmental arrest during embryogenesis and failure of pupation [28]. The results of delayed development duration and reduced food-intake, which were presented herein by using RNAi *in vivo* during the last larvae instar of *S. exigua*, indicated that *SeEcR* is necessary for appropriate developmental behavior of the insect. The effect of EcR deficiency on survival rate varied in different insects. *D.melanogaster* carrying mutations that inactivate all three isoforms of EcR are embryonically lethal [29], and *EcR* RNAi studies in *Tribolium castaneum* showed nearly 100% lethality rate in injected larvae [30]. In our study, as high as a 95% mortality prior to eclosion was observed (Fig. 3A). In addition, the *SeEcR* knockdown larvae show defective phenotypes, including the typical “half pupation” phenotype (Fig. 4E), which resembles misshaped structures that were observed after the ingestion of dsRNA for *SeCHSA* [31].

The effect of RNAi on the target gene *SeEcR* in this study was effective (Fig. 3). To confirm that the observed RNAi effect is not due to an off-target effect, a second different *SeEcR* dsRNA fragment (362 bp), designed from the DBD (DNA binding domain) domain and the D (hinge) domain of two EcR isoforms, was used for injection. Similar results were observed in survival rates (such as sharp decline during the larvae to prepupae period) and in the typical phenotypes (e.g. half pupation and abnormal eclosion) (Data not shown). This proved that the RNAi of *SeEcR* is gene-specific.

### The five genes (except *SeTre-2*) in the chitin biosynthesis pathway are regulated by SeEcR

EcR acts as an ecdysteroid-inducible transcription factor by directly interacting with USP via the DNA-response elements in the promoters of target genes [32], [33]. Apparently, the EcR plays a vital role in the transcription of multifarious genes [34], [35]. However, the role of the *EcR* in controlling the transcription of the genes responsible for the key enzymes involved in the chitin biosynthesis processes remains enigmatic. Measurement of the transcript levels following ds*EcRcom* injection allowed us to identify distinct gene-specific responses of *SeTre-1*, *SeTre-2*, *SeG6PI*, *SeUAP*, *SeCHSA* and *SeCHSB*.

Most likely, *SeEcR* continuously regulated the transcription of *SeTre-1*, *SeCHSA* and *SeCHSB* (Fig. 5A, E and F). In the bamboo borer *Omphisa fuscidentalis*, expression of *EcR-A* and *EcR-B1* was followed by a decrease in trehalose concentration and an increase in trehalase activity [36]. The *CHSA* and *CHSB* mRNA levels could be up-regulated in a stage-specific manner during the onset of metamorphosis in *D. melanogaster* [18]. Whereas, the change of *SeG6PI* and *SeUAP* transcripts in *S. exigua* were more complicated (Fig. 5C and D). The undisturbed mRNA levels of *SeG6PI* at 24 hr and an unexpectedly increased expression of *SeUAP* at 108 hr after ds*EcRcom* injection led to the hypothesis that catalyzed products of *G6PI* or *UAP* might be potential feedback inhibitory substances in the pathway. It is not suprising that the role of *UAP* and *G6PI* might be far more complex than we expected [37]. Both our studies (Figs. 5B and 6) and those of Tatun *et al*. revealed 20E and *EcR* hardly had effect on a transmembrane enzyme gene (*Tre-2*) at early detection point, which substantially dued to the uninvolvement of *Tre-2* in the dynamic changes in the hemolymph trehalose concentration that occurred during the larval–pupal transformation [38]. In short, the loss of *SeEcR* results in not only reduced abundance of the five genes transcripts responsible for the enzymatic reactions in chitin biosynthesis pathway, but also obviously suppressed chitin metabolism (Table 1) in a stage- and tissue-specific response to insect hormone during insect metamorphosis.

### The four genes in the pathway are ecdysone-regulated late genes

Ecdysone has been shown to negatively regulate transcription of pupal cuticle protein genes in imaginal disks and is required for apical extracellular matrix formation and epithelial morphogenesis in *D. melanogaster* [39]. In an *in vitro* system of lepidopteron imaginal wing discs, ecdysone had been reported to stimulate the incorporation of N-acetyl-D-glucosamine into glycoproteins, and upregulate the expression of adult cuticular genes in cell lines derived from *Tenebrio molitor* [40]. This data provides important clues in regards to an intriguing extension of the ecdysone-mediated genetic regulatory network, and the question as to whether the five genes in the chitin biosynthesis pathway are among the ecdysone-induced genes arises.

**Figure 9:**
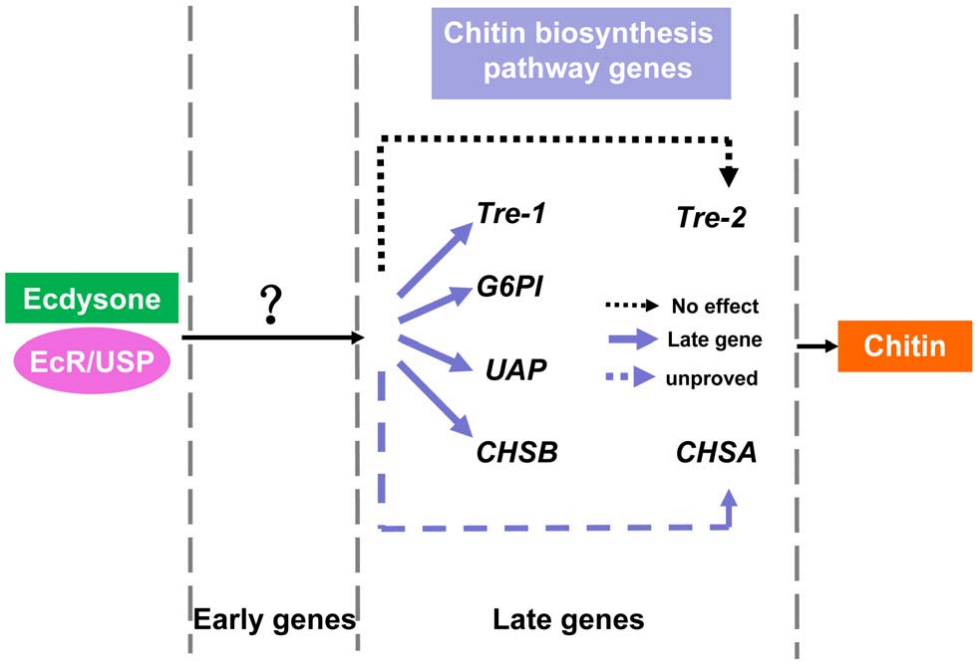
Summary of the 20-hydroxyecdysone late-response genes in the chitin biosynthesis pathway in *S.exigua*. See text for details.

Here in our study, significant up-regulation of *SeCHSA and SeCHSB* was detectable at 4 hr and durative to 36 hr after 20E injection into the larvae of *S. exigua*, and high levels of *SeTre-1, SeG6PI and SeUAP* expression were detected at 12 hr post-injection of 20E. This implies that the expression of the five genes are induced by 20E (Fig. 7). In agreement with our present results, previous *in vivo* studies had demonstrated that hemolymph trehalose concentration was sensitive to 20E level in *Omphisa fuscidentalis* [41]. *In vitro* up-regulation of *CHSA* and *CHSB* was due to an indirect interaction between the ecdysone receptor complex and ecdysone-responsive elements within the *CHSA* and *CHSB* promoters in *D. melanogaster* and these two genes act as members of the late genes [18]. The expressiong of the *mmy* gene encoding UDP-Nacetylglucosamine pyrophosphorylase (UAP) in *Drosophila* could be altered by the moulting hormone 20-Hydroxyecdysone [42]. Discriminatively, *SeTre-2* seems insensitive to 20E, which was more similar to the ecdysone-independent genes. Taken together, we speculate that the five genes *SeTre-1*, *SeTre-2*, *SeG6PI*, *SeUAP*, *SeCHSA* and *SeCHSB* excluding *SeTre-2* are among the ecdysone-induced genes hierarchies (Fig. 9).

Different 20E-responsive genes seem to be differentially regulated by ecdysone. This has been experimentally demonstrated by the *in vitro* culture experiments in larval salivary glands of *D.melanogaster* [2], [8], [9]. When there is a continuouse high level of 20E, early genes are activated with a quick response, reach their maximum expression and then regress within 4 hours. Due to the inhibition by cycloheximide (Chx) on their induced expression and a delayed reaction when incubating with ecdysone, the 20E late-response genes could be classified at a lower hierarchy position [1], [5], [43], [44]. In the *in vitro* cultured midguts, the four genes (*SeTre-1, SeG6PI, SeUAP and SeCHSB*) showed a slow peak in activation by 20E at least 4 hr after incubation, but no continuous increase in their mRNA expression was detected during the 12 hours when cultured in the presence of both Chx and 20E (Fig. 7). This response of gene transcription to 20E and Chx in this manner is characteristic of the “late” genes analogous to genes of the ecdysone regulatory hierarchy as described by Ashburner and his colleagues in *D. melanogaster* (Fig. 9).

Further studies to directly identify the relationship between the expression of the chitin biosynthesis interrelated genes and the hormone governing the stages of insect metamorphosis is currently in progress in our laboratory, and the regulation of other 20E early-response genes is required to identify in order to be verify for this connection.

## Materials and Methods

### Insects

*S.exigua* were reared in the laboratory at 25°C±2°C and 75%±5% r.h. (relative humidity) on a 14L:10D photoperiod using an artificial diet [45]. All dissections and tissue sampling of specimens were carried out in ice-cold saline and stored at −80°C for later use.

### RNA isolation and cDNA synthesis

Total RNA was extracted from individual specimens of *S. exigua* using Trizol reagent (Invitrogen, USA). Total RNA (5 µg) was used for synthesizing cDNA with the AMV reverse transcriptase XL (Takara, Japan) with the following reaction conditions: 37°C for 10 min, 42°C for 1 h, 99°C for 5 min, and 5°C for 5 min [46].

### Cloning and analysis of SeEcR cDNAs

The ABD SMART RACE cDNA amplification kit (BD Bioscience Clontech, CA, USA) was used to obtain the full-length *SeEcR* cDNA. To clone the *S. exigua* EcR gene, degenerate primers based on the conserved amino acids sequences of “PRQQEE” (forward primer, SeEcR-F) and “QPSXE” (reverse primer, SeEcR-R) were used to obtain a common fragment of EcR (Table 2). The touch-down PCR amplification reactions were carried out as follows: 94°C for 5 min, 5 cycles of 94°C for 40 s, 48°C for 40 s and 72°C for 40 s, 25 cycles of 94°C for 40 s, 54°C for 40 s and 72°C for 40 s, and with an extension step at 72°C for 10 min at the end. The amplified product was then separated on an agarose gel and purified using the Gel Extraction Kit (OMEGA, USA). The amplified fragment was subcloned into the pMD-18T vector (Takara, Japan) and sequenced. To get a longer cDNA sequence, we performed 3′-RACE and 5′-RACE using nested gene-specific primers for *SeEcR* (Table 2) and anchor primers supplied in a SMART RACE cDNA Amplification Kit (Clontech).

**Table 2:** Primers used in this study.

Information about the primers			
For dsRNA			
Name of primers		Nucleotide sequence (5′–3′)	
dsEcR-F*		**GGATCCTAATACGACTCACTATAGG** GTGTCGGTTGAAGAAATGTCT	
dsEcR-R*		**GGATCCTAATACGACTCACTATAGG** CGCAACATCATCACCTCACT	
dsEcR-SF		GTGTCGGTTGAAGAAATGTCT	
dsEcR-SR		CGCAACATCATCACCTCACT	
dsGFP-F*		**GGATCCTAATACGACTCACTATAGG** AAGGGCGAGGAGCTGTTCACCG	
dsGFP-R*		**GGATCCTAATACGACTCACTATAGG** CAGCAGGACCATGTGATCGCGC	
dsGFP-SF		AAGGGCGAGGAGCTGTTCACCG	
dsGFP-SR		CAGCAGGACCATGTGATCGCGC	
For *SeEcR* cloning			
Name of primers		Degenerate primers (5′–3′)	
SeEcR-F		CCNCGGCARCARGAGGAGC	
SeEcR-R		TCCTCBTCNGANGGCTG	
		Nested PCR primers (5′–3′)	
5-SeEcR-1		GAATCGTGGCACCACCTCGTGAAT	
5-SeEcR-2		GAGGCGGTGGATCACACTGCAT	
3-SeEcR-1		GTGTCGGTTGAAGAAATGTCT	
3-SeEcR-2		ATGCAGTGTGATCCACCGCCTC	
For quantitative real-time RT-PCR			
Gene	Forward (5′–3′)		Reverse (5′–3′)
*SeEcRcom*	GTGAGGTGATGATGTTGCGAGTA		AGAAAATGACGATGGCAGTGAG
*SeTre-1*	GGTTGGGACTTCTCAACGCGC		GACAGCATCCCGGTGATTCCC
*SeTre-2*	GGCAGGCGTCGGGATTACTTC		GGCCAGGCATTCGGATAGTCC
*SeG6PI*	ATCAGTGGACAGTGGAAAGGGTA		TTGAGGTGGTTGGCGTAAGG
*SeUAP*	CCTGATGGCAACGGAGGACTG		CAACTTTCGCCGCACAGTCAG
*SeCHSA*	GGCTCGGGTCCTCATAACGTC		GTACCTGGTCCATCGTTGGCG
*SeCHSB*	GCTCGGTTCTGCGGTTGTGTC		CCATAGCCACGTCACACGCTC

* The sequence in bond at the 5′ end of the primer is T7 promoter sequence.

On the National Center for Biotechnology Information (NCBI) website, the sequence of the two *EcR* cDNAs were compared with the other *EcR* sequences deposited in the GenBank using the “BLAST-X” tool. Amino acid sequences were deduced from the corresponding cDNA sequences using the translation tool at the ExPASy Proteomics website (http://expasy.org/tools/dna.html). A protein sequence analysis tool from the ExPASy Proteomics website (http://expasy.org/) was used to predict molecular weight. Multiple sequence alignments of the deduced amino acid sequences were made using Multiple Alignment software (http://www.ebi.ac.uk/clustalw/index.html) [47]. Phylogenic and evolutionary analyses were conducted using Molecular Evolutionary Genetics Analysis (MEGA) software version 4.1.

### dsRNA synthesis

A 546 bp fragment from the *EcR* common region (Fig. 2A) and a 714 bp fragment from *GFP* (ACY56286) were PCR amplified. dsRNA synthesis was performed using the T7 RiboMAX™ Express RNAi System Kit (Promega, USA) according to the manufacturer's instructions. Primers for *EcR* and *GFP* were designed by adding the T7 polymerase promoter sequence to their 5′ends (Table 2). The dsRNA was resuspended in an appropriate amount of nuclease-free water (DEPC water). The purified dsRNAs were quantified by spectroscopy and examined by agarose gel electrophoresis to ensure their integrity.

### RNA interference bioassays and sampling

*In vivo* RNAi in *S. exigua* larvae was performed as previously described [48]. The 2nd day of fifth-instar (the last instar) larvae were anaesthetized on ice for 2–3 min and used for the injection experiment. Five micrograms of dsRNA dissolved in 5 µl of DEPC water were injected into larvae between the second and the third abdominal segment using 10 µl microliter syringes (Hamilton), and the injection point was sealed immediately with wax. Both the dsRNA of *GFP* and an equivalent volume of buffer were used as controls.

For phenotype observation, feeding and developmental duration experiments, each group contained 30 individual larvae in each set of repetition and three replicates were used. Observation was performed every 12 hours after injection. The following three controls were included: the ds*GFP* group (an equivalent amount of *GFP* dsRNA), the buffer group (the same volume of DEPC water) and the CK group (control check with no treatment). The artificial diet pellets were cut into appropriate analogous sizes. All diets were replaced daily and each piece of food was weighed on an accurate electronic scale to measure the food intake of each individual insect. The results are expressed as the mean±standard errors (SE) of three independent replicates (n = 3), each representing 30 fifth-instar larvae. The statistical significance of data from the survival rates, food-intake and developmental duration were determined by one-way analysis of variance (ANOVA) and analyzed by Duncan multiple comparison test. Percentage data were arcsine square root transformed prior to ANOVA. For chitin content assay, each group included 50 individuals and 8 abnormal larvae were chosen for analysis (n = 8). The cuticle or midgut of individual misshapen specimens was disesscted as the chitin analysis sample. For the target gene detection experiment, each group included 60 individuals with three replicates, and the *SeEcR* mRNA levels of randomly chosen individual samples were measured at 12 hr, 24 hr, 36 hr, 72 hr and 108 hr after the injection of *SeEcR* dsRNA. For the pathway gene detection assay, each treatment included 60 individuals with three replicates and three misshapen samples were selected at 12 hr, 24 hr, 36 hr, 72 hr and 108 hr post-injection for the independent detection.

### Western blot analysis

Protein samples were made by homogenizing 1 larva per 150 µl of PBS (20 mM sodium phosphate and 130 mM NaCl, pH 7.4) plus protease inhibitor cocktail Phenylmethyl sulfonylfluoride (PMSF). After freeze thawing for three times, centrifuged at 12,000 g, 4°C for 30 min. The protein content in supernatant was determined by BCA kit, and the supernatant containing equivalent protein was mixed with 5×loading buffer for SDS polyacrylamide gel electrophoresis and boiled for 10 minutes, After centrifugation at 12,000 g for 5 min, protein samples were cooled to room temperature before loading on a 10% polyacrylamide SDS gel. The process of western blotting were modified from the method previously described [49]. A purified primary antibody (anti-SeEcRcom antibody, 1∶750 dilution) and an lgG goat anti-rabbit antibody conjugated with HRP was used for secondary antibody (BOSTER, 1∶5000 dilution).

### *In vivo* ecdysteroid injection

For the *in vivo* ecdysteroid injection experiment, 20E (Sigma, St. Louis, MO, USA) was dissolved in 5 µl of DEPC water at doses of 2 µg per insect, and each group included 30 individuals with six replicates. Three individual larvae were randomly chosen for the experiment 4 hr, 12 hr and 36 hr after injection.

### *In vitro* midgut culture experiments

The 2nd day of fifth-instar larvae were dissected in saline. The midguts together with the peritrophic membranes were ligated tightly using cotton thread at the anterior and posterior ends, and then cut to the ligatures. After the isolated midguts were rinsed four times with medium, they were independently cultured in Grace's Insect Tissue Culture Medium at 25°C±2°C for 12 hr.

Four groups were included in this culture experiments: the CK control group (control check group with no addictive in culture medium); the 20E group (added 20E hormone to final concentration of 10^−6^ M); the Chx group (added cycloheximide to a final concentration of 10^−3^ M); the 20E+Chx group (added 20E to final concentration 10^−6^ M and cycloheximide to 10^−3^ M). Each group included 10 individual tissues with three replicates. Samples for mRNA expression analysis were selected randomly at 0 hr, 4 hr, 8 hr and 12 hr after incubation.

### Quantitative real-time RT-PCR

The relative mRNA expression of genes was assessed by quantitative real-time reverse transcription polymerase chain reaction (qRT-PCR) by using the SYBR Green based detection system (SYBR Premix Ex Taq, Takara, Japan) with LightCycler480 system (Roche, Germany). This assay allows for precise quantification of weakly expressed genes. The oligonucleotides used in qRT-PCR are indicated in Table 2. Specificity of the reaction was checked by analysis of the melting curve of the final amplified product. The housekeeping gene and target gene from each sample were run in parallel on the same PCR plate. All of the qRT-PCR reactions were performed using the following common program: pre-incubation at 95°C for 10 s, followed by 45 cycles of denaturation at 95°C for 15 s, annealing at 60°C for 20 s, and elongation at 72°C for 15 s. At the end of each cycle, a fluorescence reading were used to determined the extent of amplification. Standard curves were obtained using a 10-fold serial dilution of pooled cDNA from all stages.

As described above, cDNA was synthesized from specimens of *S. exigua* from experimental group and the *GFP* dsRNA group at every detection point using the AMV reverse transcriptase XL (Takara, Japan). The qRT-PCR measurement was performed in triplicate and normalized to an internal control for each sample (the *S. exigua* β-actin gene AY507963, Table 2). The relative expression level of the target gene was calculated by the mRNA level in the treated group divided by the level in the control group.

### Chitin analysis

A modified analysis procedure was used in the chitin content measurement experiment as previously described [50]. For chitinase digestion, each pellet was resuspended in 250 µl of McIlvaine's buffer, and *Streptomyces plicatus* chitinase (Sigma, 5 mg/ml in PBS, 50 µg for cuticle and 25 µg for midgut) was added and incubated for 72 h at 37°C to hydolyse chitin to GlcNAc. Standard curves were generated for data processing.
